# Mechanism of antagonist ligand binding to REV-ERBα

**DOI:** 10.1038/s41598-024-58945-4

**Published:** 2024-04-10

**Authors:** Mohammad Homaidur Rahman, Lamees Hegazy

**Affiliations:** 1 Center for Clinical Pharmacology, Washington University School of Medicine, University of Health Sciences and Pharmacy, St. Louis, MO USA; 2 Department of Pharmaceutical and Administrative Sciences, University of Health Sciences and Pharmacy, St. Louis, MO USA

**Keywords:** Biochemistry, Biophysics, Computational biology and bioinformatics

## Abstract

REV-ERBα, a therapeutically promising nuclear hormone receptor, plays a crucial role in regulating various physiological processes such as the circadian clock, inflammation, and metabolism. However, the availability of chemical probes to investigate the pharmacology of this receptor is limited, with SR8278 being the only identified synthetic antagonist. Moreover, no X-ray crystal structures are currently available that demonstrate the binding of REV-ERBα to antagonist ligands. This lack of structural information impedes the development of targeted therapeutics. To address this issue, we employed Gaussian accelerated molecular dynamics (GaMD) simulations to investigate the binding pathway of SR8278 to REV-ERBα. For comparison, we also used GaMD to observe the ligand binding process of STL1267, for which an X-ray structure is available. GaMD simulations successfully captured the binding of both ligands to the receptor’s orthosteric site and predicted the ligand binding pathway and important amino acid residues involved in the antagonist SR8278 binding. This study highlights the effectiveness of GaMD in investigating protein–ligand interactions, particularly in the context of drug recognition for nuclear hormone receptors.

## Introduction

Nuclear hormone receptors (NRs) constitute a superfamily of transcription factors that regulate gene transcription in response to various stimuli and control a myriad of biological processes^1–3^. Examples of well-known NRs are vitamin D receptor, retinoic acid receptor, and peroxisome proliferator-activated receptor. Nuclear hormone receptors (NRs) represent a major drug target, accounting for ~ 16% of all approved drugs^4^. NRs have been targeted successfully in many therapeutic areas, including diabetes, skin disorders and breast and prostate cancers^5–7^.

NRs are characterized by multiple domain organization. Two of these domains are highly conserved and contribute to the activation of NRs: i. DNA-binding domain (DBD), located at the N terminus, known as activation function 1 (AF-1). DBD is ligand independent and interacts with target DNA sequences through two zinc fingers to recognize specific hormone response elements (HREs), ii. the Ligand binding domain (LBD), which is located at the C-terminal domain and interacts with small molecule ligands and cofactors involved in regulating the transcription, known as AF-2^8^. The LBD is a globular domain composed almost exclusively of α-helices arranged as three layers “sandwich shape”. Ligands bind to the ligand binding pocket (LBP) within the interior of this globular domain and depending on the nature of the ligand, a conformational change of the LBD occurs leading to a cascade of downstream events^9–13^.

NRs assume a wide range of conformational states, including apo states, ligand specific states and recent studies implicate an important role for protein dynamics in the mechanism of action of nuclear receptor ligands^15–17^. REV-ERBα and REV-ERBβ (en-coded by NR1D1 and NR2D2, respectively) are a subfamily of the nuclear receptors (NRs). REV-ERB influences processes such as gluconeogenesis and lipid metabolism and modulating REV-ERBα activity has shown promise in preclinical studies for the treatment of metabolic disorders such as obesity, type 2 diabetes, and dyslipidemia^14,15^. Several studies demonstrated that REV-ERB activation can effectively suppress the transcription of IL-1β and NLRP3, two genes that are implicated in the regulation of inflammatory pathways^16,17^. Additional research suggested that activating REV-ERBα could serve as a novel pharmacological strategy for treating inflammatory pain^18^. REV-ERBs also play key role in the circadian clock regulation, influencing the expression of genes involved in various physiological processes, including metabolism, immune function, and the sleep–wake cycle. Dysregulation of circadian rhythm has been associated with various health conditions, including metabolic disorders, cardiovascular diseases, and certain cancers^19–21^. Recent studies highlighted their role in neurological disorders such as cognitive diseases^22^. Although REV-ERBs share similar molecular domain organization to most nuclear hormone receptors, they are distinctive because they lack the carboxy-terminal helix-12 activation function 2 (AF-2) region. Therefore, they are effective transcription repressors and interact constitutively with the NRs corepressors such as NCoR via helix-11 of the C-terminal ligand-binding domain^23,24^. Upon binding to the DNA response element, REV-ERBs recruit corepressors to the target gene causing its repression through active histone deacetylation and condensation of the chromatin^24,25^.

**Figure 1:**
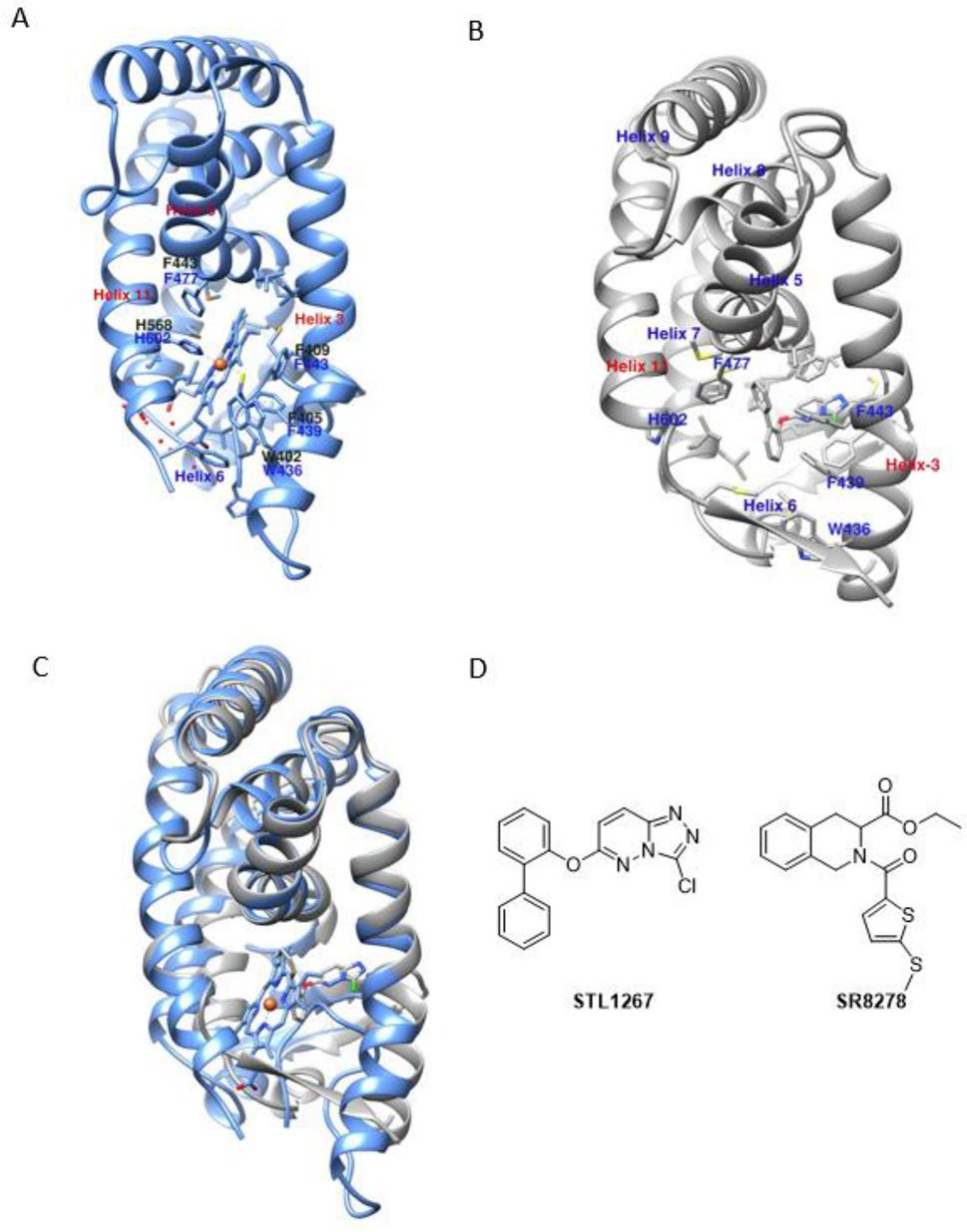
(**A**) X-ray structure of heme bound REV-ERBβ (PDB:6WMQ). (**B**) X-ray structure of REV-ERBα bound with the synthetic agonist STL1267 (PDB:8D8I). (**C**) Overlay of REV-ERBβ/heme (Protein colored in blue ribbons and heme represented as blue sticks) and REV-ERBα/STL1267 (Protein colored in grey ribbons and STL1267 represented as grey sticks) (**D**) 2D representation of agonist STL1267 and antagonist SR8278.

X-ray crystal structures are invaluable tools in drug development, providing detailed information that facilitates rational drug design and optimization. Heme is the natural ligand for REV-ERB, and the X-ray structures of REV-ERB bound to heme and corepressor peptide have been reported^26^. Recently, the first X-ray structure of REV-ERBα bound to a synthetic agonist ligand (STL1267) and corepressor peptide has been determined^27^. Although Both agonists effectively bind to the NCoR CoRNR box ID1 peptide, the conformation of REV-ERB LBD (ligand-binding domain) in both structures shows significant differences, particularly in the helix-3, helix-6 and helix-11 regions (RMSD values of ligand binding pocket regions after fitting the backbone atoms of both structures are 2.9 Å, 2.7 Å and 2.6 Å for helix-3 residues, 430–440, helix-6 residues 505–513, C-terminal helix-11 residues 605–611 using the residue numbering in PDB: 8D8I) (Fig. 1). While heme and STL1267 partially occupy similar regions within the LBP (ligand binding pocket) of REV-ERBα, heme occupies a region that is closer to helix-11 and the corepressor binding surface and is partially exposed to the solvent. On the other hand, STL1267 binds to a deeply buried hydrophobic pocket within the LBD, which is located closer to helix-3 (Fig. 1C). The interactions between STL1267 and the residues within the LBP are predominantly hydrophobic in nature. The provided information suggests that the ligand binding pocket (LBP) of REV-ERB is flexible and can accommodate various ligand scaffolds^19^.

There is a scarcity of chemical tools available to study the biology of REV-ERB, with only a few ligands being utilized in the past decade. Among them, SR8278 stands as the sole antagonist that can be used to investigate the function of REV-ERB in different disease models (Fig. 1D)^28^. Pharmacological inhibition of REV-ERBα have been shown to protect dystrophic muscle from injury by promoting myogenic repair, and therefore may have therapeutic utilities for muscular dystrophy^20^. However, SR8278 has limitations in terms of low metabolic stability and a short half-life. This highlights the importance of identifying additional antagonists to explore the therapeutic potential of REV-ERB inhibition. While in silico screening methods like pharmacophore modeling and molecular docking of chemical libraries could be beneficial for uncovering novel REV-ERB antagonists, the lack of X-ray structures depicting the binding of REV-ERB to SR8278 has impeded these endeavors^21–26^. Obtaining structural information for nuclear hormone receptors poses several challenges due to their high degree of their conformational flexibility^27,28^. Therefore, we decided to investigate the ligand binding pathway for this system. Classical molecular dynamics simulations (CMD) are valuable tools for studying protein dynamics and ligand binding interactions^29–32^. However, important conformational changes , including ligand binding events, occur on timescales that may take milliseconds to seconds, that are longer than what is feasible for traditional CMD simulations that are limited to tens of microseconds^33–35^. This limitation makes it challenging to capture the complete binding pathway within the simulation time. To tackle this challenge, biasing simulation techniques have proven valuable in improving sampling and calculating free energy of biomolecules. These methods include umbrella sampling^36,37^ metadynamics^38,39^, conformational flooding^40^ adaptive biasing force (ABF) calculations^41,42^, etc. Gaussian accelerated molecular dynamics (GaMD), can surmount timescale constraints by overcoming energy barriers. They enable a more extensive exploration of energy landscapes, offering valuable insights into the processes of ligand binding^43–47^. Therefore, we employed Gaussian accelerated molecular dynamics (GaMD) to predict the ligand binding pathway of the REV-ERB antagonist SR8278. Additionally, we also used GaMD to observe the ligand binding process of STL1267 as a control simulation since the X-ray structure of REV-ERBα bound with STL1267 is available. Our GaMD simulations effectively demonstrated the binding of at the receptor’s orthosteric site. GaMD enhanced sampling simulations captured the binding pathway of both these ligands to the orthosteric pocket of human REV-ERBα. Prior to conducting this study, it was unclear where SR8278 binds, whether it binds to the orthosteric site or an allosteric site. Therefore, this study provides insights into the mechanism of antagonist ligand recognition by the REV-ERBα receptor, ultimately facilitating the rational design of drugs targeting REV-ERBα.

## Results

### GaMD simulations captures a STL1267 bound pose that closely resembles the X-ray structure

**Figure 2:**
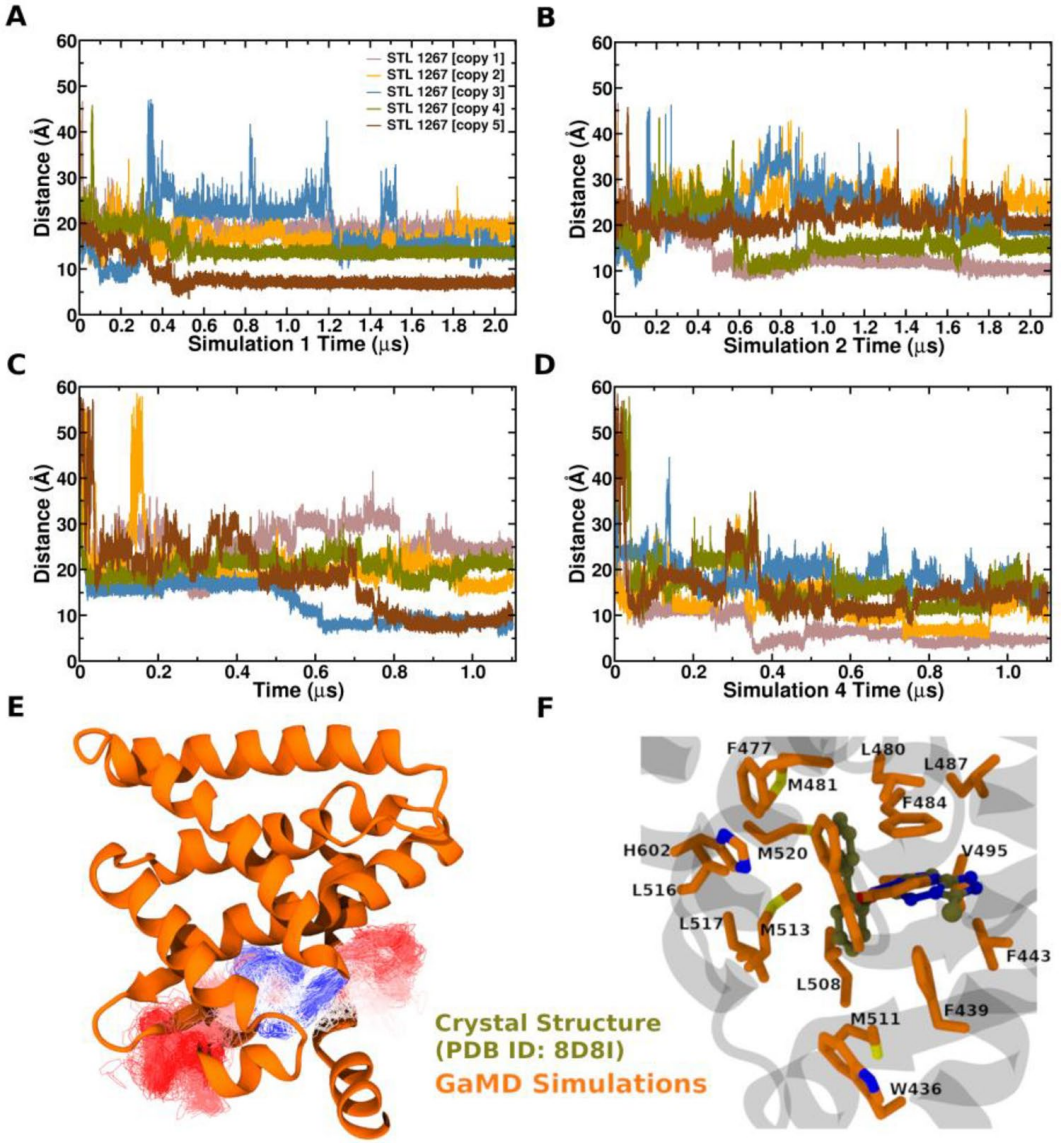
Center-of-mass (COM) distance between STL1267 agonist ligand heavy atoms and Cα atoms of amino acid residues: Phe484, Phe497, and Leu517 in the GaMD simulations trajectories of (**A**) Lig1Sim1, (**B**) Lig1Sim2, (**C**) Lig1Sim3, and (**D**) Lig1Sim4. (**E**) The ligand binding pathway observed for “Lig1Sim3” and “Lig1Sim4”, for which the STL1267 is represented by lines and colored by simulation time in a red–white–blue (RWB) color scale. The red color indicate beginning of simulation and blue represent end of simulation time. (**F**) Comparison of 3D conformation of ligand STL 1267 crystal bound PDB ID: 8D8I to GaMD simulation.

We initiated four independent GaMD simulations with five unbound STL1267 molecules (Table 1), placed randomly at a distance of 20–50 Å from the protein surface (Supplementary Fig. S1A). In all GaMD simulations, we observed the entry of a single STL1267 molecule toward the ligand binding pocket of REV-ERBα (Fig. 2). However, in two of the simulations, specifically Lig1Sim1 and Lig1Sim4, we observed the STL1267 ligand moving deeper into the orthosteric binding pocket. This observation is confirmed by a decrease in the center of mass distance between the ligand’s heavy atoms and the Cα atoms of orthosteric pocket residues Phe484, Phe497, and Leu517 over the course of the simulation, indicating the ligand’s penetration into the orthosteric pocket of REV-ERBα (Fig. 2A,D). The ligand was observed accessing the orthosteric pocket through either the co-repressor binding site or through the region closer to the β-sheets (Fig. 2E), and at approximately 500 ns, it has penetrated further into the orthosteric pocket and assumed a binding pose that closely matches the X-ray structure-bound pose (Fig. 2F). Our analysis of hydrogen bonds suggested that hydrogen bonding interactions along the association pathway play an important role in ligand binding to the orthosteric site. In Lig1Sim1, ligand copy 5 and Lig1Sim2, ligand copy 1, the STL1267 ligand moved from the bulk solvent toward the orthosteric pocket, initiated by hydrogen bonding interactions with Arg448 on helix-5. As STL1267 entered more deeply into LBP, it formed a hydrogen bonding interaction with Ser608 or Phe609 and subsequently with His609 on helix-11. These interactions, coupled with hydrophobic interactions, provided stability to the binding pose of STL1267 within the orthosteric pocket (Supplementary Fig. S2a). However, although the STL1267 molecule in Lig1Sim2, ligand copy 1 and Lig1Sim3, ligand copy 3 formed hydrogen bonding interactions with several amino acid residues on helix5 and helix11, the ligand didn’t move deeper in to the orthosteric pocket (Supplementary Fig. S2b). In Lig1Sim4, ligand copy 1, the STL1267 ligand moved from the bulk solvent toward the orthosteric pocket, initiated by hydrogen bonding interaction with Gln506 on the β-sheets region and Gln493 on helix-6. Even though GaMD simulations effectively identified the binding site for STL1267, the triazolo-pyridazine group of STL1267 in the predicted pose adopts a flipped orientation, as opposed to its ligand binding pose in the X-ray structure (Fig. 2E). The biphenyl groups of STL1267 maintained similar orientation to the X-ray crystal structure. These results suggests that while GaMD simulations can successfully predict the binding site and pose of ligands to REV-ERB, they may not consistently reproduce the accurate ligand binding conformation. The ligand is stabilized mainly by hydrophobic interactions, particularly via π-π interactions involving Phe439, Phe443, Phe477 and Phe484 (Supplementary Fig. S3).

**Table 1:** Type and duration of GaMD Simulations performed in this study.

Name ID	Ligand	Duration (ns)
Lig1Sim1	STL1267	2050
Lig1Sim2	STL1267	2050
Lig1Sim3	STL1267	1050
Lig1Sim4	STL1267	1050
Lig2Sim1	SR8278	1050
Lig2Sim2	SR8278	2050
Lig2Sim3	SR8278	1050
Lig2Sim4	SR8278	1050
Lig2Sim5	SR8278	2050
Lig2Sim6	SR8278	1050
Lig2Sim7	SR8278	1050

### GaMD simulations of the antagonist SR8278

**Figure 3:**
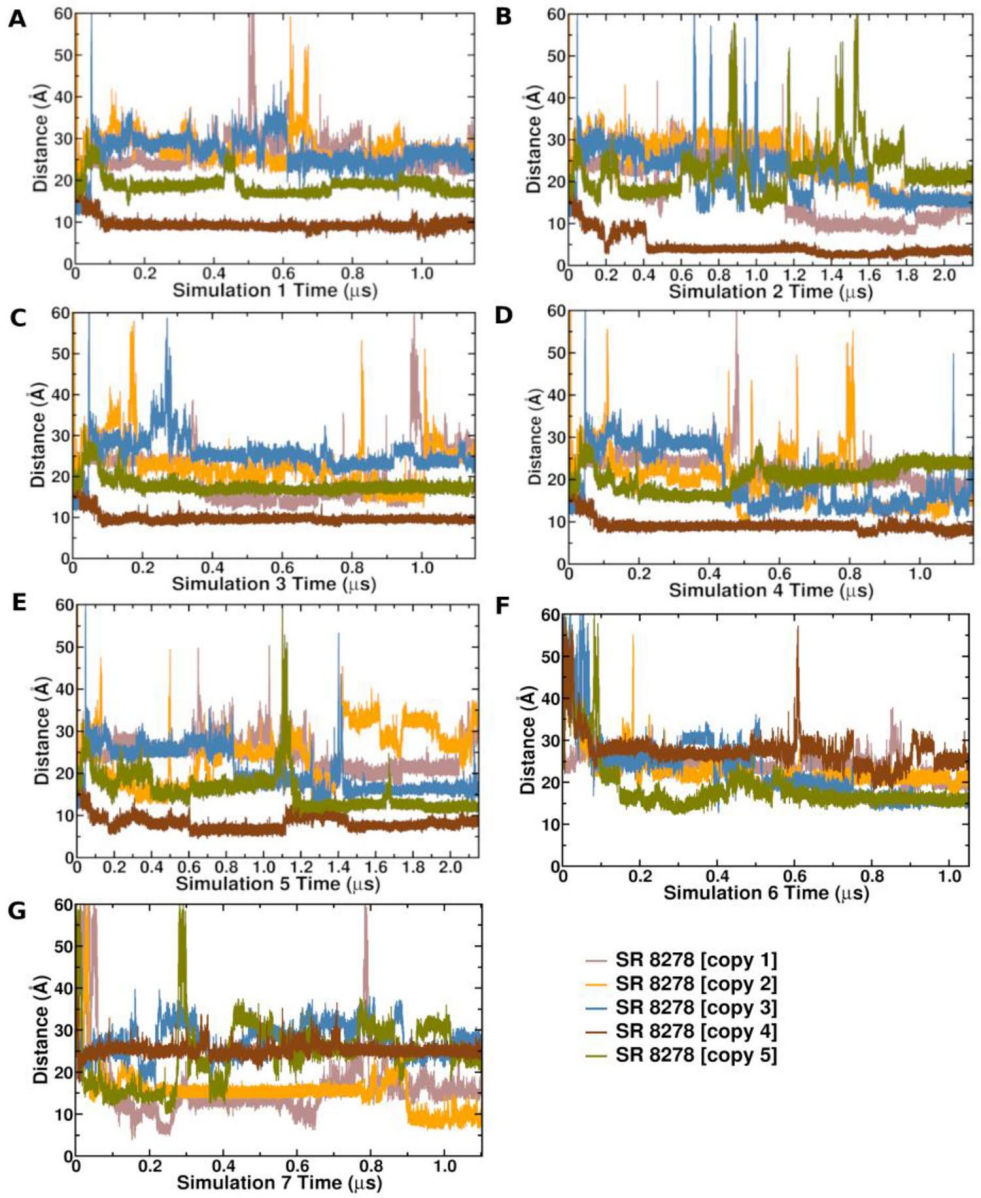
Center-of-mass (COM) distance between SR8278 antagonist ligand heavy atoms and Cα atoms of amino acid residues: Phe484, Phe497, and Leu517 in GaMD simulations (**A**) Lig2Sim1, (**B**) Lig2Sim2, (**C**) Lig2Sim3, (**D**) Lig2Sim4, (**E**) Lig2Sim5, (**F**) Lig2Sim6, and (**G**) Lig2Sim7.

**Figure 4:**
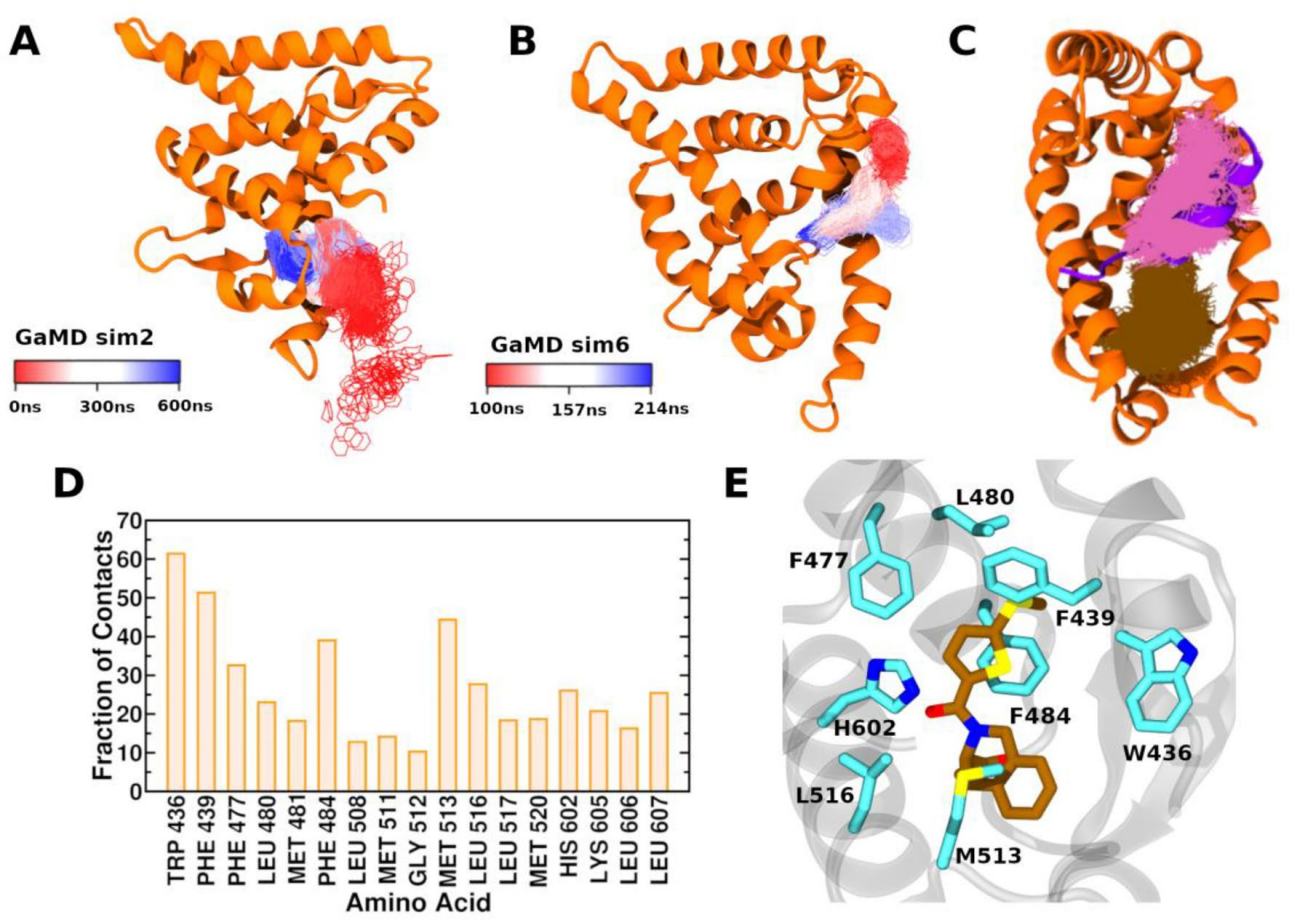
The visual representation of ligand binding pathway observed in (**A**) “Sim2” and (**B**) “Sim6”, for which the center ring of SR8278 is represented by lines and colored by simulation time in a red–white–blue (RWB) color scale. (**C**) Snapshots of SR8278 ligand frames in GaMD simulations. Ligand frames binding at the orthosteric pocket are represented as brown sticks. Ligand frames binding at the corepressor binding site are represented as pink sticks. The protein ribbons are represented as orange ribbons and the NCoR-ID1 corepressor peptide is shown as purple ribbons (PDB: 6WMQ). The trajectory frames are presented at 100-ps time intervals. Ligands not bound to the orthosteric or the NCoR-ID1 corepressor sites are hidden for clear visualization. (**C**). Fraction of contact between the bound ligand and protein from ~ 7 µs GaMD simulations. (**D**) Close view of orthosteric pocket amino acid residues establish the most significant interactions with the antagonist SR8278. Hydrogen atoms are hidden for clear visualization.

With GaMD simulations accurately predicting the ligand binding site of STL1267, aligning closely with the X-ray structure (Fig. 2F), we conducted seven distinct Gaussian accelerated molecular dynamics simulations (GaMD) to investigate the binding pathway of the antagonist SR8278 to REV-ERBα (Table 1). In five GaMD simulations (Lig2Sim1, Lig2Sim2, Lig3Sim3, Lig4Sim4, Lig2Sim5), five SR8278 molecules were placed in the solvent at a minimum distance of 15–20 Å from the protein. In two other GaMD simulations (Lig2Sim6 and Lig2Sim7), five SR8278 molecules were placed randomly in the solvent at a distance of 40–50 Å from the protein surface. The protein stability was assessed in all simulations as evidenced by the root mean square deviation of the protein backbone atoms and per residue root mean square deviation (Supplementary Figs. S4 and S5). In all five GaMD simulations (Lig2Sim1, Lig2Sim2, Lig3Sim3, Lig4Sim4, Lig2Sim5) where the ligand molecules were placed ~ 20 Å from the protein, we observed the binding of one SR8278 ligand molecule to the orthosteric pocket in each simulations (Fig. 3). In the GaMD simulation, Lig2Sim6, one of the ligand molecules was observed binding on the coreprssor binding surface but didn’t move deeper inside the orthosteric pocket (Fig. 4B). Ligand binding was not observed in Lig2Sim7. Ligand binding was verified by monitoring the distance over simulation time between the ligand’s center of mass (COM distance) and three specific amino acid residues within the ligand binding pocket (Phe484, Phe497, and Leu517) (Fig. 3). The root mean square fluctuations calculations (RMSF) further revealed that the N-terminal regions of helix-3, C-terminal region of helix-11, helix-6, and the loop regions connecting helix-3 and helix-4 and helix-8, helix-9 and helix-10 were the most flexible regions of the protein (Supplementary Fig. S4B). Across all five simulations (Lig2Sim1, Lig2Sim2, Lig3Sim3, Lig4Sim4, Lig2Sim5), one of the ligand molecules consistently relocated from the bulk solvent to the interface between helix-3 and helix-6 of the receptor, eventually entering the orthosteric site of the receptor (Fig. 4A). Throughout the manuscript, our emphasis will be mainly on the ligand copy that binds to the orthosteric site of the receptor (SR8278 copy 4, Fig. 3).

In order to gain further insight into the SR8278 ligand’s binding mechanism, we analyzed its hydrogen bonding interactions in each simulation. In all the GaMD simulations, the antagonist SR8278 initially formed a hydrogen bond with the amino group of Lys605 side chain located on the C-terminal region on helix-11 (Supplementary Fig. S6). The ligand formed different patterns of hydrogen bonding interactions in every simulation, indicating that the SR8278 antagonist ligand exhibited different binding orientations inside the orthosteric pocket. In simulations Lig2Sim2, Lig2Sim4 and Lig2Sim5, the ligand formed hydrogen bond interaction with the side chain of His602 while this interaction was absent in simulations Lig2Sim1 and Lig2Sim3. In simulations Lig2Sim4 and Lig2Sim5, the ligand is engaged in a hydrogen bond interaction with the side chain of Trp436, situated on Helix 3. The Trp436-SR8278 hydrogen bond interaction was partially observed in simulation Lig2Sim1 and Lig2Sim3 and not observed in simulation Lig2Sim2.

To determine the most important amino acid residues involved in ligand binding, we calculated the fraction of contacts of protein amino acid residues within 6.0 Å of the bound ligand for the combined simulations trajectories (Fig. 4D). The ligand is involved in hydrophobic contacts with Trp436 located on helix-3 for 60% of the combined trajectory. The ligand also makes considerable hydrophobic interactions with Phe439 located also on helix-3, Phe477, Phe484 located on helix-5, Met513, Leu516 located on helix-7 and His602 located on helix-11 (Fig. 4E).

We then calculated the dihedral angle distribution for amino acid residues within the ligand binding pocket (Supplementary Fig. S7). Notably, the dihedral angle of Trp436 (C-CA-CB-CG) exhibits distinct behavior prior to and after ligand binding, while the N-CA-CB-CG dihedral angle of Trp436 maintains a value of 60° before ligand binding, it assumes multiple values following ligand binding. Similar behavior is observed for Phe477 and His602, indicating an increase in the flexibility of their side chains during the SR8278 binding.

**Figure 5:**
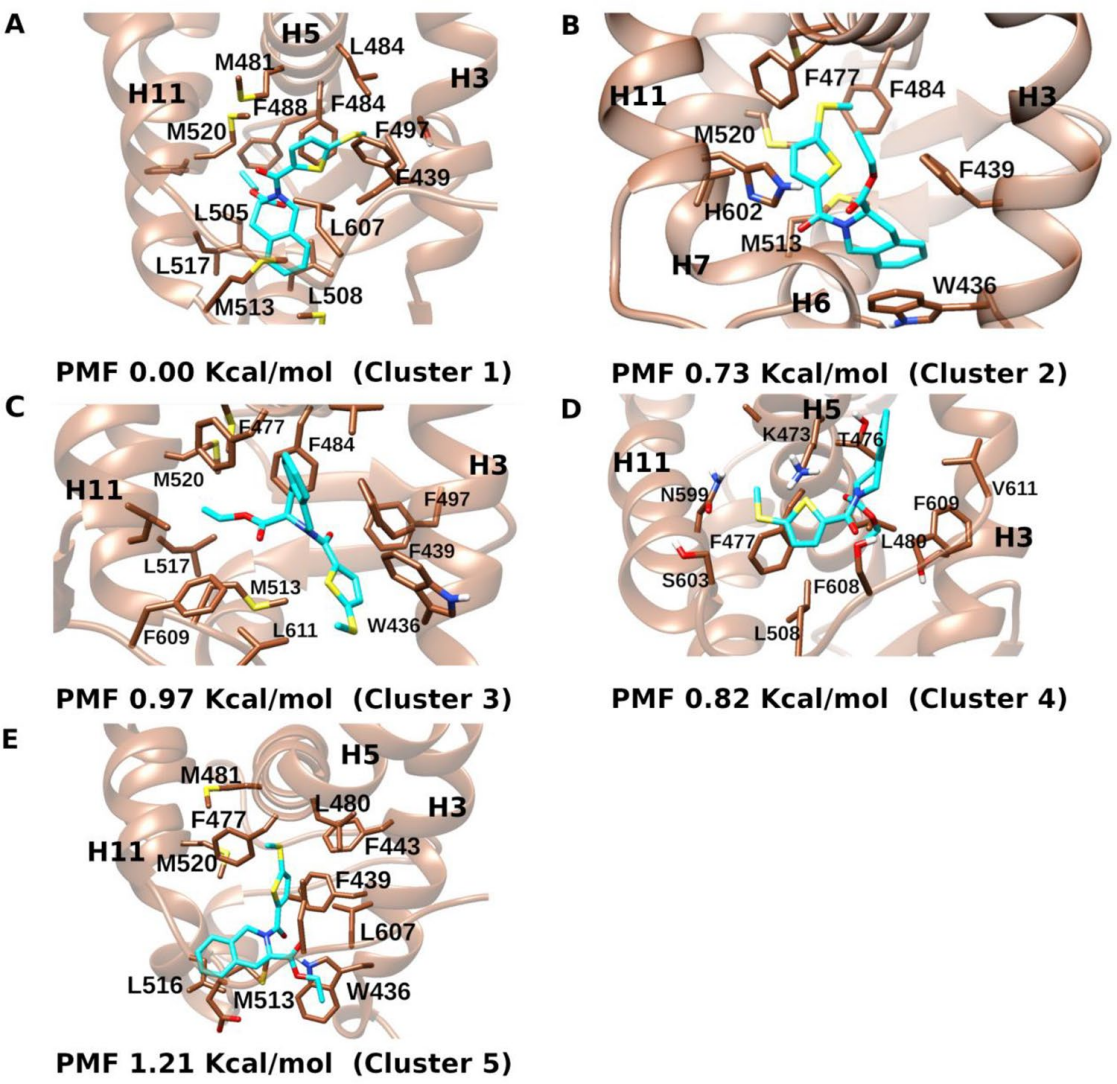
Molecular interactions of the top five ligand clusters and their corresponding PMF value, calculated from combined GaMD simulations trajectories.

Next, we performed ligand RMSD-based structural clustering for the combined ligands trajectories to identify distinct poses of the antagonist ligand binding to REV-ERBα. Figure 5 displays the top five clusters with the highest population, along with their respective free energies. Notably, four of these clusters are located within the orthosteric pocket, each exhibiting distinct orientations of the ligand (Fig. 5). Cluster 4 depicted the binding of the ligand within a cavity on the surface of the receptor, which corresponds to the corepressor binding site (Supplementary Fig. S7). Interestingly, throughout all the simulations, an additional instance of the ligand was consistently observed to bind to the corepressor binding site (Fig. 4C). This observation suggests that the corepressor binding site could potentially serve as a meta-stable binding site for REV-ERB ligands. The most populated cluster (cluster 1, 0.0 kcal/mol) corresponds to a ligand binding pose where the tetrahydro isoquinoline (THIQ) occupies the heme binding region, closer to helix-6 and the C-terminal region of helix-11 and is making hydrophobic contacts with Met513, Leu517, Leu508 and Leu607. The ethyl ester group is oriented towards helix-7 while the methyl thiothiophene group is oriented towards helix-3 (Cluster 1, Fig. 5). In cluster 2 (0.73 kcal/mol), the ligand occupies both the heme and STL1267 binding regions (Fig. 5). The THIQ group is oriented towards helix-3 and is involved in π–π stacking interactions with Trp436 (Cluster 2, Fig. 5). The methyl thiothiophene group is oriented towards helix-11. The carbonyl group is involved in hydrogen bonding interaction with His602. In cluster 3 (0.97 kcal/mol), the ligand orientation is flipped with the THIQ orientation towards helix-5, involved in π–π stacking interactions with Phe439 and the methyl thiothiophene group is oriented towards helix-3, occupying a cavity in the orthosteric pocket that is not occupied by either heme or STL1267 and making hydrophobic contacts with Trp436 (Cluster 3, Fig. 5). In cluster 4 (0.82 kcal/mol), the ligand is not bound in the orthosteric pocket but rather is bound on a hydrophobic site on the protein surface that corresponds to the corepressor binding site (Cluster 4, Fig. 5) where it is involved in hydrophobic interactions with Leu608, Phe609 and aliphatic side chains of Ser603, Lys473 and Asn599. In cluster 5 (1.21 kcal/mol), The ligand occupies mainly the heme binding region, with THIQ group oriented towards the C-terminal region of helix-11 and both the ester and the methyl thiothiophene groups are oriented towards helix-3 (Fig. 5).

## Discussion

GaMD captured complete binding of the agonist STL1267 and antagonist SR8278 to the orthosteric pocket of REV-ERBα. In the STL1267 simulations, GaMD simulations captured a ligand binding pose closely similar to the X-ray binding pose (Fig. 2F). Consequently, we explored the ligand binding pathway for SR82787, the only antagonist for REV-ERBα and for which no X-ray crystal structure is available. SR8278 binding was captured by GaMD simulations within ~ 100 ns in five independent simulations to the orthosteric pocket. STL1267 and SR8278 explored distinct ligand binding pathways in the GaMD simulations. STL1267 is observed to access the orthosteric pocket through the co-repressor binding site, or through the region between helix-6 and the β-sheets (Fig. 2E). On the other hand, SR8278 diffused from the solvent to the protein orthosteric pocket through interface formed by helix-3 and helix-6 (Fig. 4C). SR8278 initially made hydrogen bonding interaction with the side chain of Lys605 during its entry to the ligand binding pocket (LBP) followed by hydrogen and hydrophobic interactions with amino acid residues inside the LBP which further stabilized the bound ligand (Fig. 4 and Supplementary Fig. S6). Analysis of the fraction of native contacts revealed that the bound ligand interacted with the amino acid residue Trp436 for more than 60% of the simulation time. Additionally, the ligand formed significant hydrophobic interactions with Trp436, Phe439, Phe477, Phe484 and Met513. The identified binding pose of SR8278 aligns very closely to the binding pose of heme (Supplementary Fig. S7). The simulations indicated that another copy of SR8278 and STL1267 bind to the corepressor binding groove which might be a metastable binding site (Fig. 4C). While it is generally understood that multiple binding and unbinding events are required for accurate computation of converged ligand binding free energy, the structural clustering of the GaMD simulations enabled the identification of energetically preferred distinct binding poses of the ligand, with the lowest energy cluster of SR8278 being identified in the orthosteric site, aligning well with the heme bound pose (Fig. 5).

Overall, these findings demonstrate the utility of GaMD in studying ligand binding to nuclear hormone receptors. The effective reproduction of experimentally observed ligand binding sites in GaMD simulations proves the method’s credibility in studying protein–ligand interactions and applicability to diverse ligands, making it suitable for studying different classes of compounds that may bind to nuclear hormone receptors. This flexibility allows researchers to investigate the interactions of various ligands with the receptor, providing a comprehensive understanding of the ligand-binding landscape. This knowledge will play a pivotal role in guiding drug discovery efforts targeting this receptor.

## Materials and methods

The X-ray crystal structure of REV-ERBα protein bound with agonist STL1267 (PDB ID: 8D8I) was taken as a starting structure for running GaMD simulations^48^. The missing residues Gly409-Gly429 were modeled using the online Swissmodel server^49^, and the structural stability of modeled structure was confirmed by running 100 ns conventional molecular dynamics simulations (CMD) of REV-ERBα protein with the agonist STL1267. For the GaMD simulations, the starting conformation of REV-ERBα protein was prepared by removing the co-repressor and STL1267 from the last frame of the CMD simulation. Then, five STL1267 agonist or SR8278 antagonist ligands molecules were randomly placed in a simulation box at 18–50 Å from the orthosteric pocket residue Phe484, Phe497, and Leu517. The Tleap module was used to neutralize and solvate the complex using explicit TIP3P water extending up to 15 Å from the surface of the protein in a cubic periodic box. All the interaction parameters for the STL1267 and SR8278 ligands were assigned according to the general AMBER force field (GAFF2)^50,51^ and AM1BCC coulombic charges were assigned to the ligand using Antechamber modules^52^ of AmbrTools23. The FF14SB forcefield parameters were used for all receptor residues^53^. The water solvated REV-ERBα–ligand complexes were first energy minimized using steepest descent (2500 steps) and conjugate gradient methods (2500 steps). Then the system was gradually heated with the Langevin thermostat from 0 to 300 K over 30 ps at constant volume using a 1 fs time step and using collision frequency 2 ps^−1^. Initial velocities were sampled from the Boltzmann distribution while keeping weak restraints on the heavy atoms of the solute and the ligand. Each system was then equilibrated in the isothermal-isobaric ensemble (NPT) at 300 K using a constant pressure periodic boundary with an average pressure of 1 atm. Isotropic position scaling was used to maintain the pressure with a relaxation time of 2 ps using Berendsen barostat. For all simulation the 10 Å cut off was chosen for nonbonded interactions, and the long-range electrostatic interactions were computed using the particle mesh Ewald (PME) method. The SHAKE algorithm was used to keep bonds involving H atoms at their equilibrium length.

Production simulation was performed by solving Newton’s equations using 2 fs time step. GaMD simulations are performed using the GaMD module implemented in the graphics processing unit (GPU) version of AMBER2022^54^. Before GaMD simulation, the system is equilibrated for 100 ns using NPT ensemble using same protcol as discuss above without any restraints. Then GaMD acceleration potential parameters are collected over 65 ns equilibration after adding the boost potential, and finally five independent GaMD production runs were performed in the NVT ensemble with randomized initial atomic velocities. The systems used for GaMD simulations are given in Table 1. All GaMD simulations were run at the “dual-boost”: one boost potential was applied to the dihedral energetic term and another to the total potential energetic term. The threshold energy was set to lower bound E = Vmax. The average and standard deviation (SD) of the system potential energies were calculated every 200,000 steps (400 ps). The upper limit of the boost potential SD, σ0 was set to 6.0 kcal/mol for both the dihedral and the total potential energetic terms. VMD (Version 1.9.4a57, http://www.ks.uiuc.edu/Research/vmd/vmd-new/devel.html) and Xmgrace (Version 5.1.25, https://launchpad.net/ubuntu/focal/+package/grace) softwares were used to make figures and plots, respectively^55^. CPPTRAJ and Chimera were used to analyze the GaMD simulation trajectories^56,57^. For clustering, a hierarchical agglomerative (bottom-up) approach was used to cluster the protein–ligand conformations using ligand-RMSD. The hierarchical agglomerative algorithm method is implemented in CPPTRAJ of AmberTools23. All the GaMD trajectories were aligned on the first frame of GaMD Simulations and then ligand-RMSD was calculated using protein backbone as reference co-ordinate without fitting the co-ordinate. The average linkage distance method is used to separate each member of cluster by using the epsilon value 1.0 and the trajectory is sieved at a stride of 500 frame. The output data of frame vs cluster is utilized to calculate the potential of mean force (PMF) value of each cluster using the PyReweighting toolkit. PyReweighting utilizes cluster and GaMD boost weight applied for each conformation to reweight Cluster PMF. Further details of the reweighting technique can be found here: https://github.com/MiaoLab20/pyreweighting.

### Supplementary Information

Supplementary Video 1.

Supplementary Information.

Supplementary Information.

## Data Availability

The Molecular Dynamics (MD) simulation initial coordinates, input files and coordinate files of the top five clusters for the GaMD Simulations of the Antagonist SR8278 can be found in the following GitHub repository: https://github.com/mohammadhrahman/GaMD_Simulations_Input/tree/main.
