# Supplementary material for: Mechanism of antagonist ligand binding to REV-ERBα
Source: Sci Rep. 2024 Apr 10;14:8401. doi: 10.1038/s41598-024-58945-4 (PMC11006950; doi:10.1038/s41598-024-58945-4)
Supplement: Supplementary file 2 — Supplementary Information. [file 41598_2024_58945_MOESM2_ESM.docx]

Supplementary Material

**Mechanism of Antagonist Ligand Binding to REV-ERBα**

Mohammad Homaidur Rahman^1,2^, Lamees Hegazy^1,2^*

^1^Center for Clinical Pharmacology, Washington University School of Medicine, University of Health Sciences and Pharmacy, St. Louis, MO

^2^Department of Pharmaceutical and Administrative Sciences, University of Health Sciences & Pharmacy, St. Louis, MO

*** Correspondence:**
Corresponding Author
lamees.hegazy@uhsp.edu


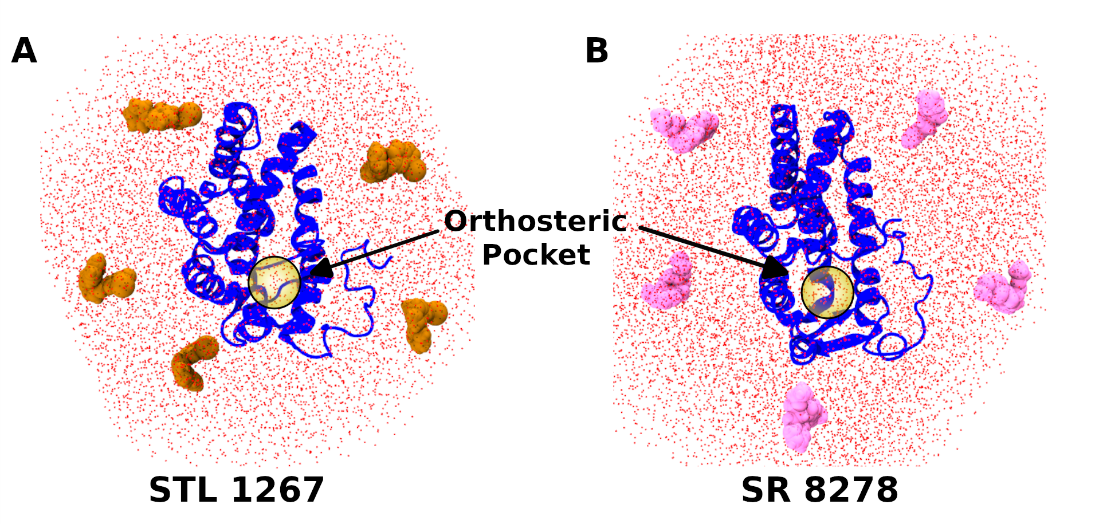


Supplementary Figure S1. Visual representation of initial conformation used in the GaMD simulations. The protein REV-ERBα is shown as blue ribbons and five copies of ligand (a) STL 1267, (b) SR 8278 are shown in sphere representation. Red dots represent water oxygen.


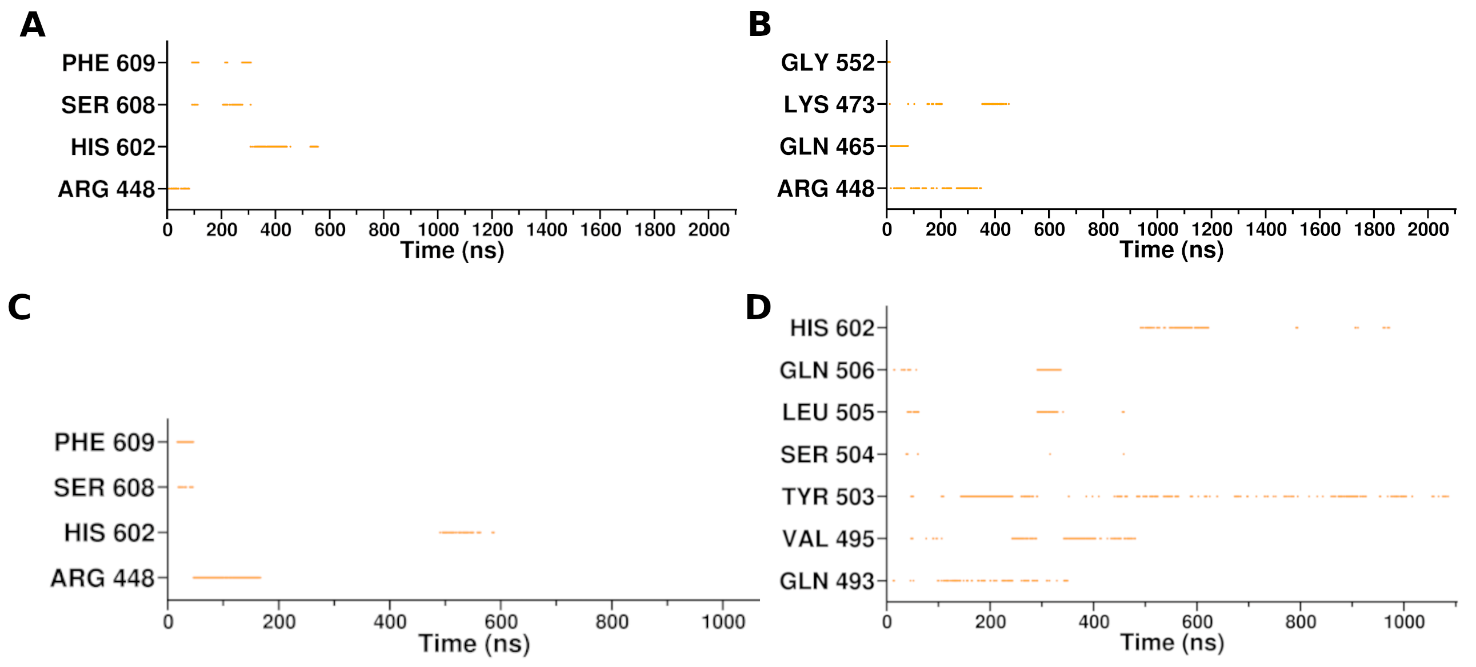


**Supplementary Figure S2**. Time evolved hydrogen bonds interactions between the agonist STL1267 and REV-ERBα amino acid residues in the GaMD simulations trajectories (A) Lig1Sim1, (B) Lig1Sim2, (C) Lig1Sim3, and (D) Lig1Sim4.


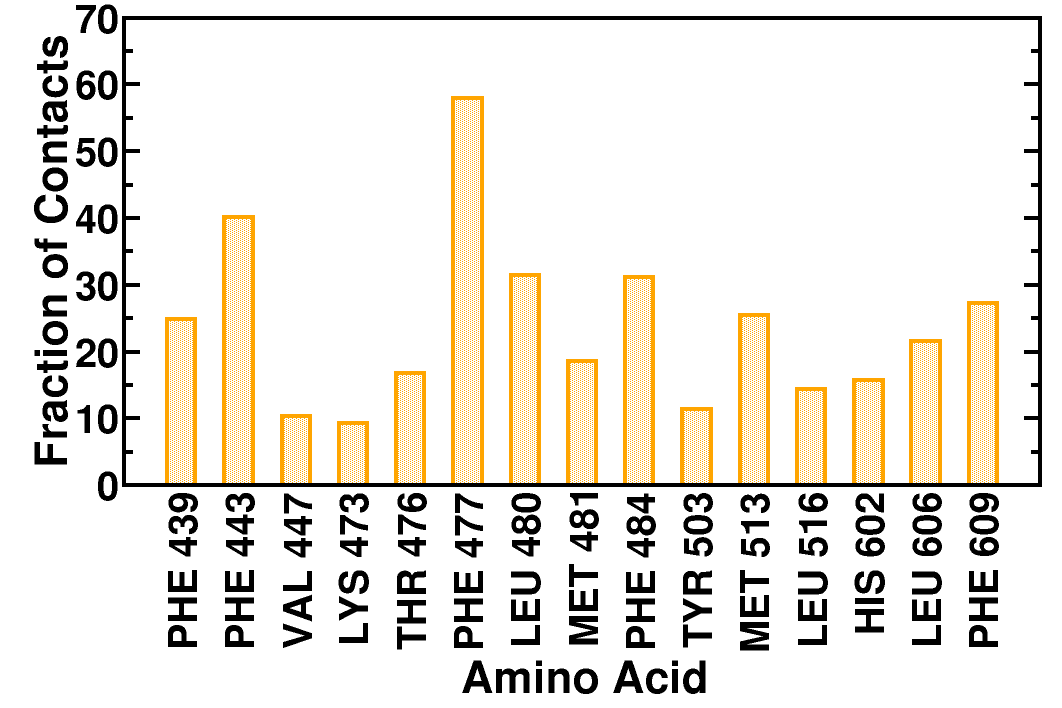


**Supplementary Figure S3**. Fraction of contact between the bound STL1267 ligand and REV-ERBα amino acid residues from the combined ~6µs GaMD simulations trajectories, Lig1Sim1, Lig1Sim2, Lig1Sim3, and Lig1Sim4.

**A**


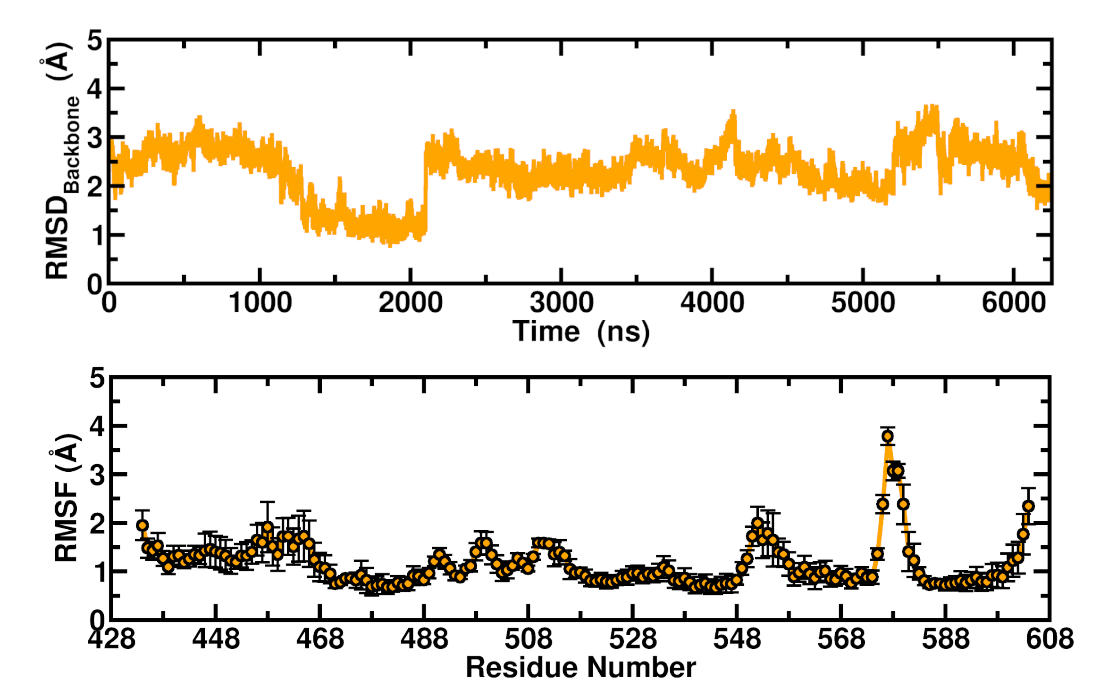


**B**


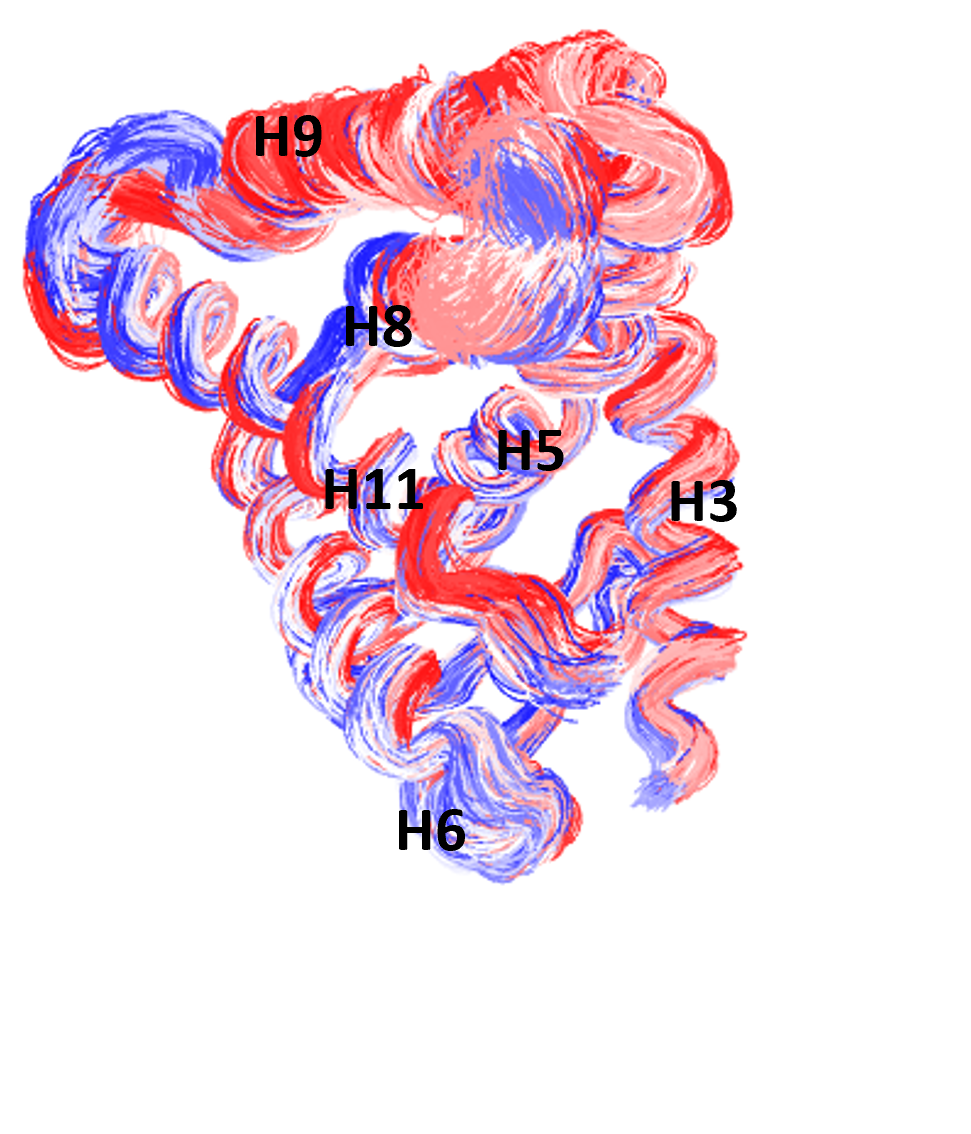


**Supplementary Figure S4.** (A) All the GaMD trajectories were combined to calculate root-mean-square deviations (RMSDs) and root-mean-square fluctuations (RMSF) (for residues Gln433-Glu604) using protein backbone atoms. (B) An overlay of multiple conformations of REV-ERB from GaMD Simulation 2 trajectories at intervals of 100 ps. The RED-White-Blue color indicates the start and end times for the trajectory.


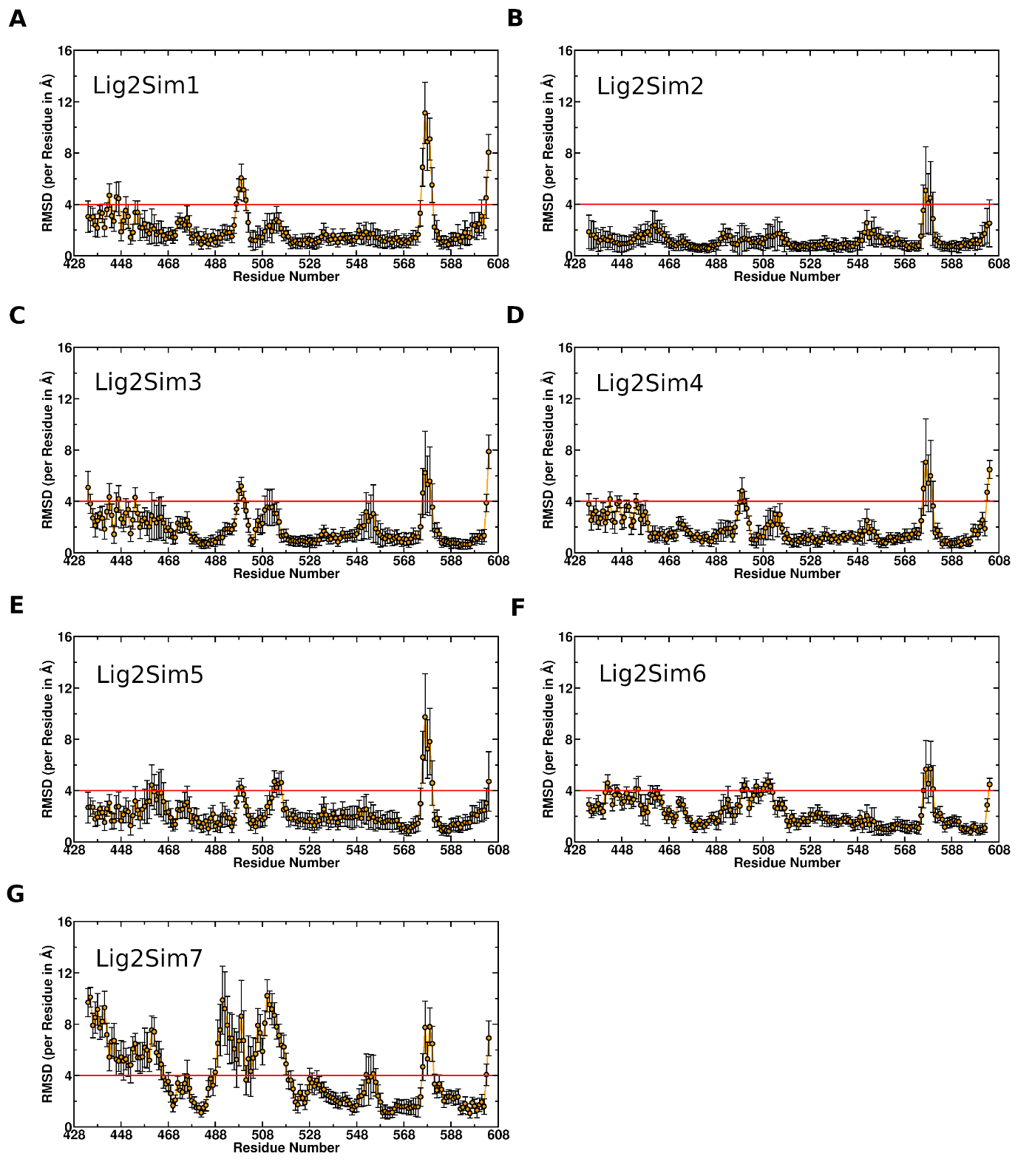


**Supplementary Figure S5.** Per residue root-mean-square deviations (RMSDs) of each amino acid of REV-ERBα protein using Cα atoms for residues 433-604 in (A) Lig2Sim1, (B) Lig2Sim2, (C) Lig2Sim3, (D) Lig2Sim4, (E) Lig2Sim5, (F) Lig2Sim6, and (G) Lig2Sim7. The mean and standard deviation data point for each Cα atom are obtained over the simulation frames of the GaMD simulation trajectories.

 
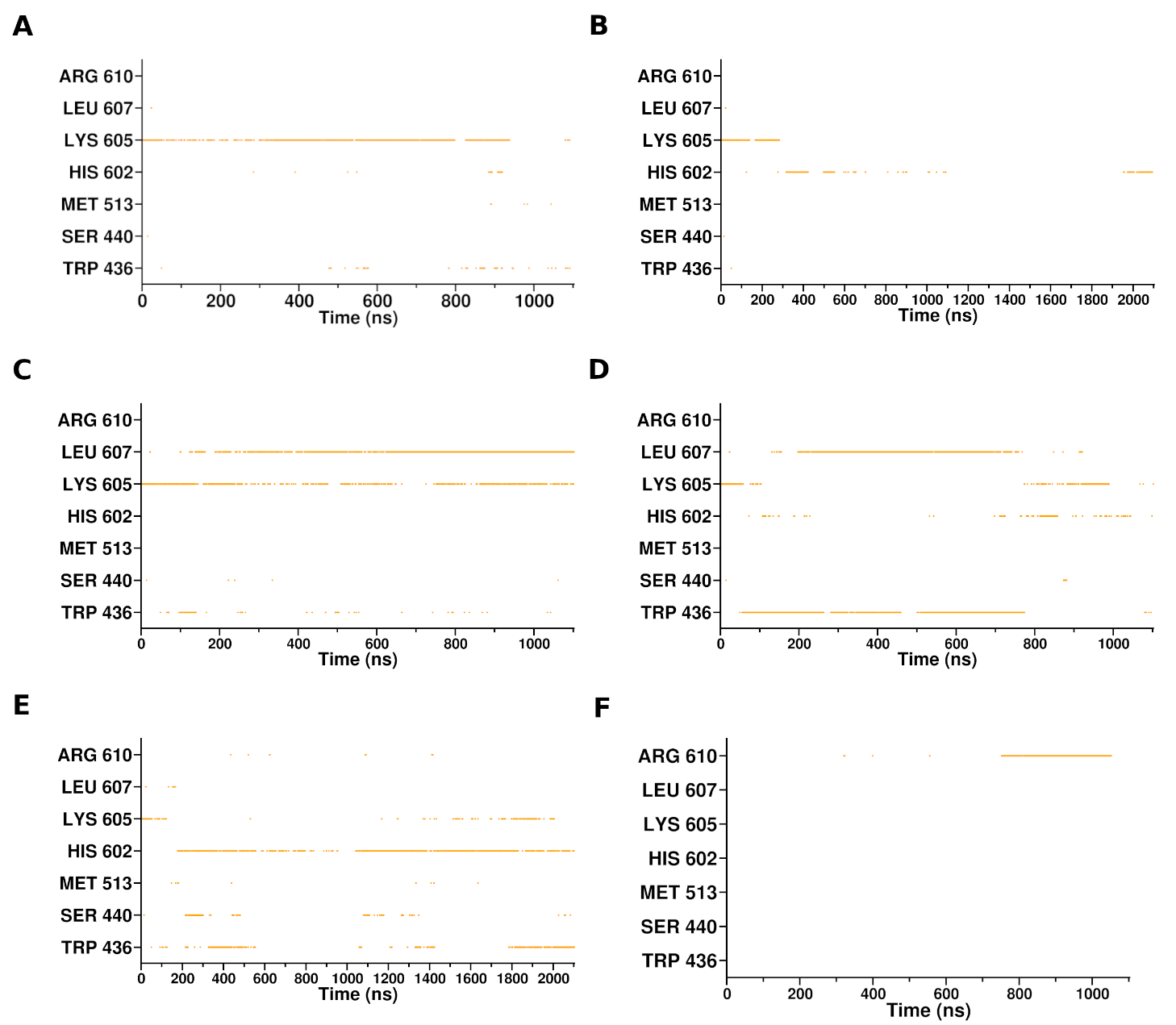


**Supplementary Figure S6**. Time evolved hydrogen bonds interactions between the antagonist SR8278 and REV-ERBα amino acid residues in GaMD simulations trajectories: (A) Lig2Sim1, (B) Lig2Sim2, (C) Lig2Sim3, (D) Lig2Sim4, (E) Lig2Sim5, and (F) Lig2Sim6.


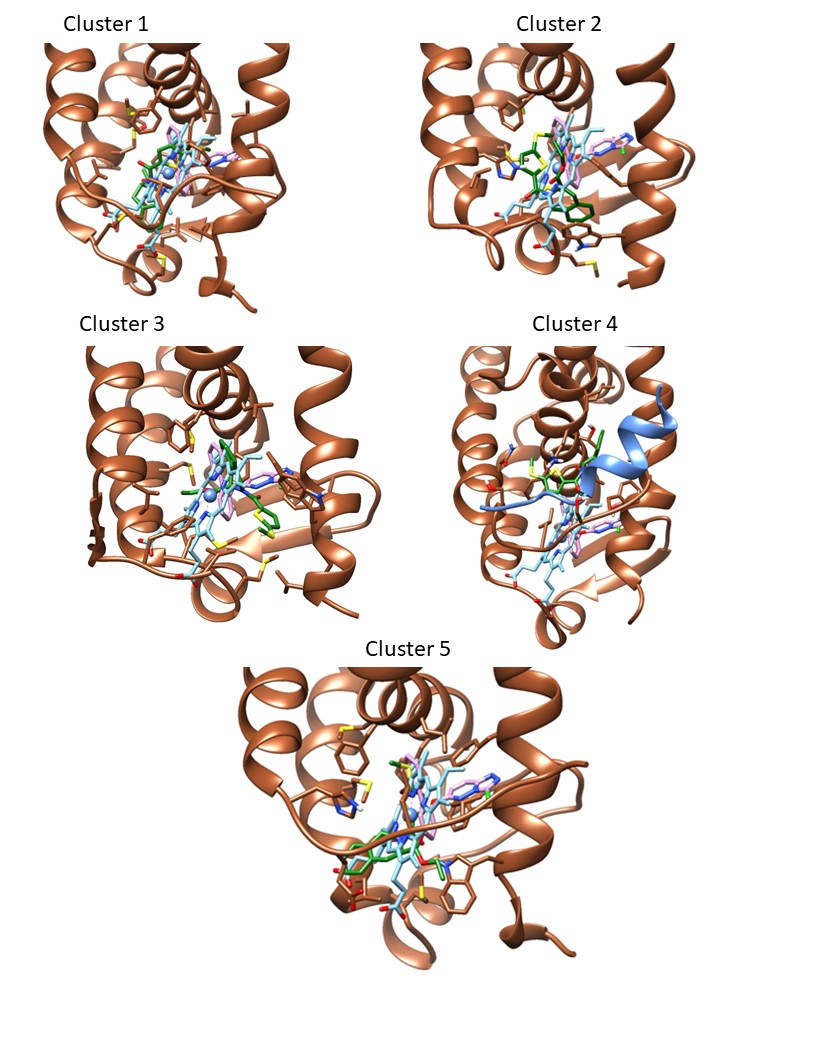


**Supplementary Figure S7.** Conformational representations of the top five populated SR8278 clusters obtained from GaMD simulation using the hierarchical agglomerative algorithm. The SR8278 binding pose is compared with the heme binding pose (blue stick bonds, PDB: 6WMQ) and the synthetic agonist STL1267 binding pose (pink stick bonds, PDB: 8D8I), respectively. In cluster 4, SR8278 was observed binding at the corepressor site (blue ribbons, PDB: 6WMQ).


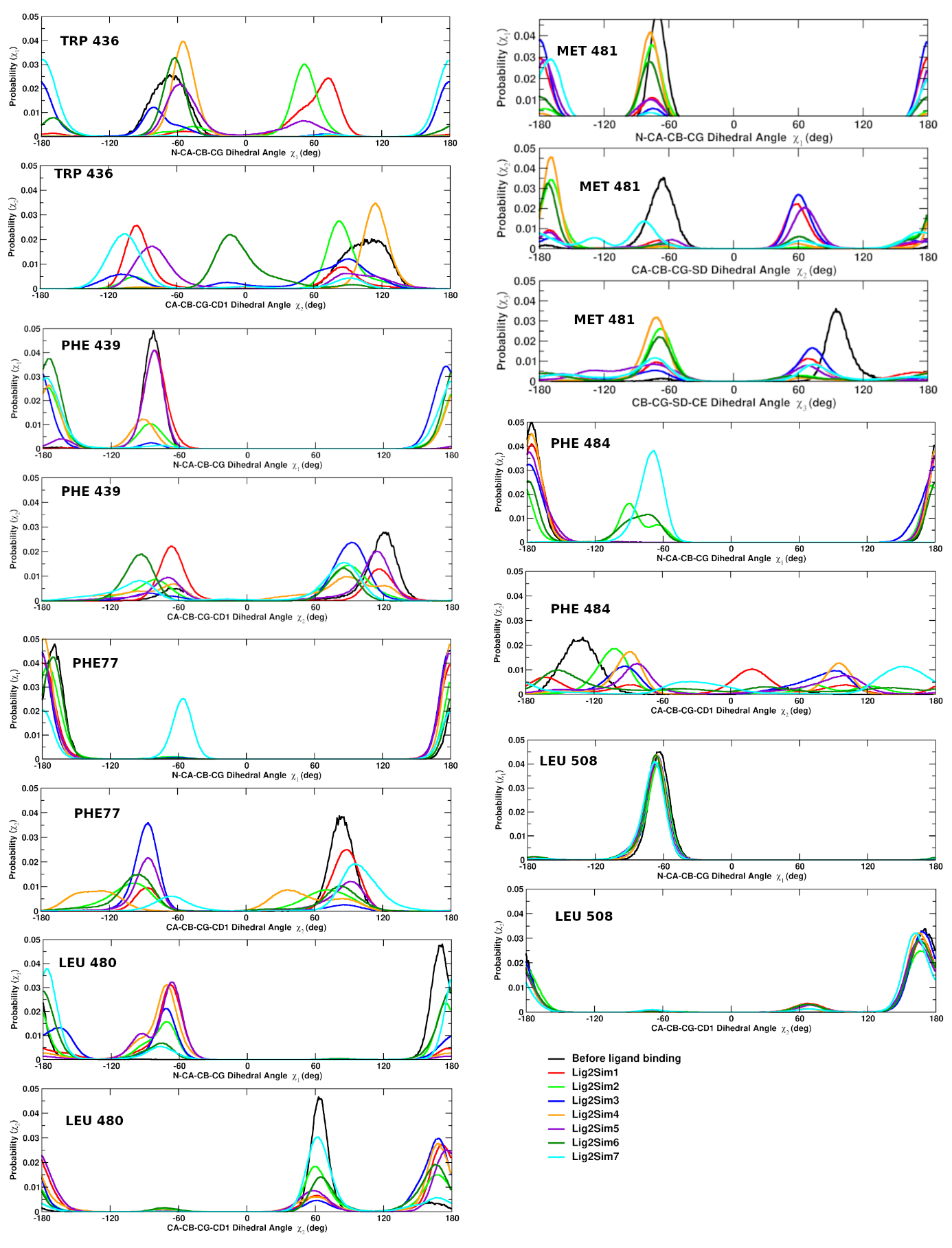


Supplementary Figure S8. Histograms of dihedral angle distributions of amino acid residues inside the ligand binding pocket before and after ligand binding.


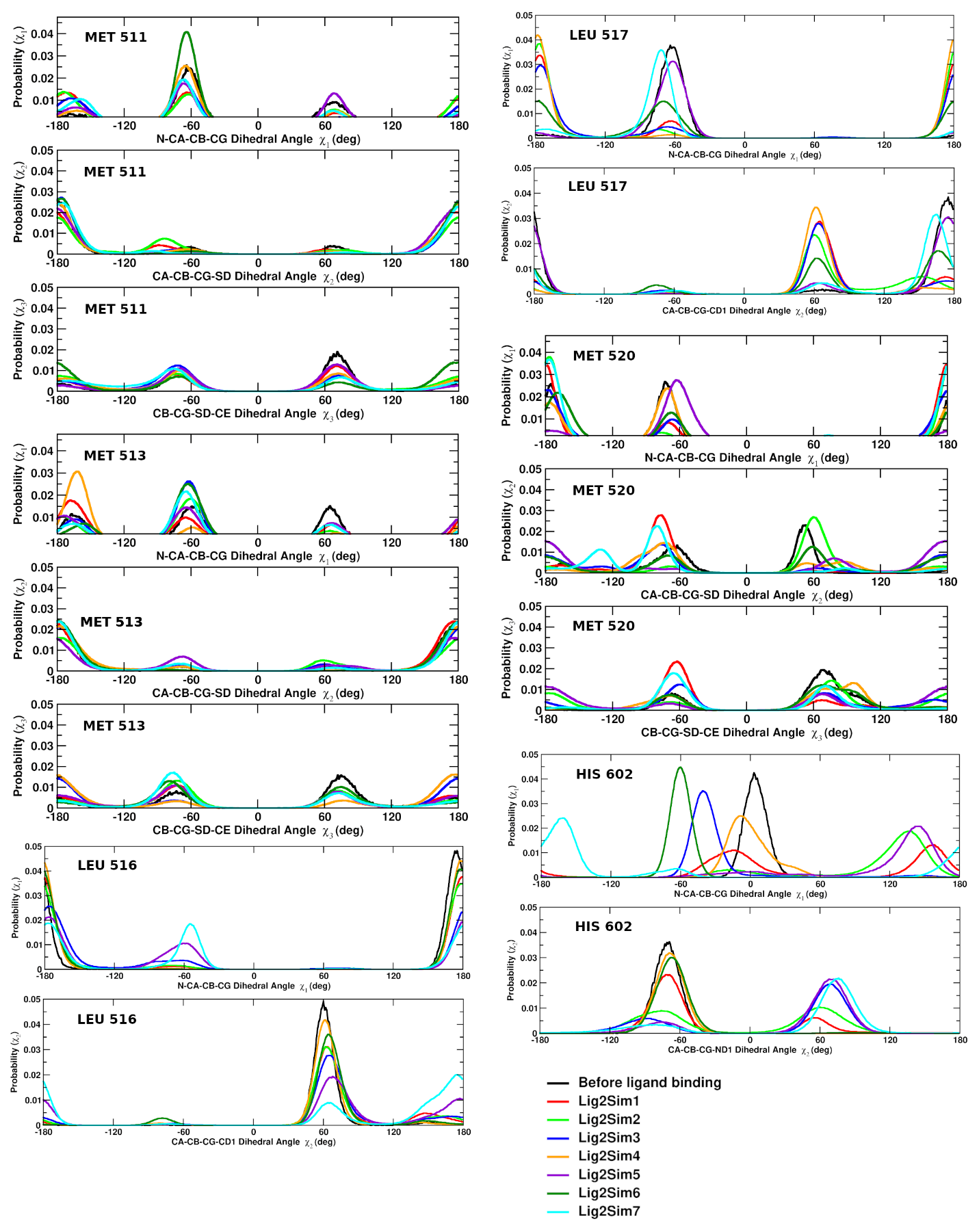
Supplementary Figure S8. Cont.

**Supplementary video.** Additional video content showcasing the binding trajectories of SR8278 observed in simulation 2 (Lig2Sim2). The video is provided as a separate file under the name:

REVERB_Binding_Sim2.mp4
