# Supplementary material for: Mechanism of antagonist ligand binding to REV-ERBα
Source: Sci Rep. 2024 Apr 10;14:8401. doi: 10.1038/s41598-024-58945-4 (PMC11006950; doi:10.1038/s41598-024-58945-4)
Supplement: Supplementary file 3 — Supplementary Information. [file 41598_2024_58945_MOESM3_ESM.docx]

**Supplementary Materials:**

Figure of the initial model used in the GaMD simulations.; RMSD and RMSF plots of protein backbone atoms.; Time course of hydrogen bonding interactions of the ligand and the orthosteric pocket amino acid residues; Histograms of dihedral angle distributions of amino acid residues inside the ligand binding pocket before and after ligand binding.; Location of top five populated ligand clusters in the GaMD simulations; movie of SR8278 binding pathway-Lig2Sim2.mp4.
